# Depression: point-prevalence and risk factors in a North Cyprus household adult cross-sectional study

**DOI:** 10.1186/s12888-017-1548-z

**Published:** 2017-12-04

**Authors:** Mehmet Çakıcı, Özlem Gökçe, Asra Babayiğit, Ebru Çakıcı, Ayhan Eş

**Affiliations:** 1 Department of Psychology, Near East University, Arts and Science Faculty, Lefkosa-Kibris, Mersin 10, Turkey; 2 Department of Psychological Counselling and Guidance, Near East University, Faculty of Education, Lefkosa-Kibris, Mersin 10, Turkey

**Keywords:** Prevalence, Major depression, Risk factors, Socio-cultural risk factors

## Abstract

**Background:**

Depression is one of the most common diagnosed psychiatric disorders in the world. Besides individual risk factors, it is also found that environment and socio-cultural factors are the other main risk factors for depression. In this article, the results of the 2016 national household survey of depression in North Cyprus (NC) are presented. The aim of the study is to determine the prevalence and possible risk factors of depression in NC households.

**Methods:**

The study was conducted between April and June 2016, the sample consisting of Turkish-speaking individuals between 18 and 88 years of age living permanently in NC. A multi-stage stratified (randomized) quota was used in the survey, and 978 people were selected according to the 2011 census. A 21 item questionnaire prepared by the researchers and a Turkish version of the Beck Depression Inventory scales were used for obtaining data.

**Results:**

This cross-sectional study found a point prevalence of 23.4% for relatively high BDI scores (≥17) suggesting clinical depression. Being female, a widow, unemployed, having a limited education and low income level, having a physical illness, living alone, and using illicit substances were defined as possible risk factors for depression.

**Conclusions:**

When we consider the world prevalence, NC has one of the higher depression prevalence. NC has environmental and socio-cultural characteristics such as a history of war, migration and colonization, high unemployment rates, socioeconomic problems, similar to other extremely high prevalence depression countries and regions, which give a strong indication of the importance of socio-cultural factors on depression.

**Electronic supplementary material:**

The online version of this article (10.1186/s12888-017-1548-z) contains supplementary material, which is available to authorized users.

## Background

Depression is one of the most commonly diagnosed psychiatric disorders and is ranked as the 4th main form of disability in the world [1]. More than 350 million people are affected by depression worldwide [2]. A lifetime prevalence study of 38,000 people in 10 countries identified proportions varying between 1.5-19.0% [3]. The highest depression proportions are found in the Middle East, North Africa, sub-Saharan Africa, Eastern Europe and the Caribbean. The lowest proportions were in East Asia, followed by Australia/New Zealand and Southeast Asia, especially in Japan [4]. In Africa the point prevalence also displayed high proportions: 22.6% for women and 14.3% for men [5]. The prevalence of major depressive disorders in Western European countries meanwhile is around 5% [6]. Interestingly though, in neighbouring countries high prevalence is evident. Clinical frequency for depression in Turkey is 10% while point prevalence is 13-20% [7]. In research applied in the south part of Cyprus among Greek Cypriots, the point prevalence of depression was found to be 27.9% amongst 1500 college students [8].

According to previous studies, one of the main possible risk factors for depression is genetic features which cause functional and structural changes in the brain [9]. Consequently, children whose family members have suffered depression have a higher proportion of major depression. [10]. Silberg, Maes and Eaves (2010) used an expansive twin sample to determine the genetic and environmental correlates of parental and childhood depression [11]. In much research throughout the world, being female increases the risk of depression when compared with males [12, 13]. Another factor which influences depression is marital status. While depression can be observed more frequently in widowed and divorced people, it is less likely to seen in married people [14, 15]. The risk of developing depression is higher for people who are above the age 55 at which point thoughts of death and medical problems increase [16, 17]. Onsets of major attacks of depression are also related to stressful life events [18]. Traumatic events such as shock caused by the death of a loved one, serious illness, or domestic violence can all increase the tendency to depression [19, 20]. Further, it has also been identified that social environment and financial problems are other possible risk factors for depression [21].

This study is the first household survey study conducted to find out the prevalence and possible risk factors of major depression in North Cyprus (NC). North Cyprus population has a history of war, migration, economic hardship and traumatic events. The presence of the Major Depression and the risk factors that increase the likelihood of occurrence may be as diverse as the similarities between the populations. Besides the basic risk factors effecting depression, socio-cultural factors involve additional risks affecting the level of prevalence. Differences in socio-cultural structure may bear additional risk factors as well as they can influence and cause differentiation of basic risk factors from community to community. Determining the socio-cultural structure and common characteristics in some specific regions of the world, such as the NC, will enable the risk factors of depression to be studied in a wider perspective and to become more aware of the socio-cultural characteristics. Although there has been growing curiosity regarding depression and its effects on the NC population limited reliable information is available regarding Major Depression. Hence, the main aims of this study are to provide a scientific analysis of the possible risk factors of depression in NC, and to provide a characterization of all the dimensions of major depression that emerge from the study.

## Methods

### Sampling

The population of the study is Turkish-speaking individuals between 18 and 88 years of age living in North Cyprus. A multi-stage stratified (randomized) quota was used to achieve a representative sample of the adult population in the survey, and 994 people were selected for household interview. The sample size was calculated by sampling formula of known population (n = Nt2pq/d2 (N-1) + t2pq) [22] where n was the sample size; N was the Population size; and t was the value for selected alpha level of 0.025 in each tail = 2.58 (the alpha level indicates the level of risk the researcher is willing to take that true margin of error that may exceed the acceptable margin of error). Besides, (p)(q) was the estimate of variance which was 0.25 (maximum possible proportion 0.5) * 1 (maximum possible proportion 0.5) which produce maximum possible sample size. Finally, d was the acceptable margin of error for proportion being estimated at 0.05 (error a researcher is willing to accept).The selected participants were tabulated according to gender (male/female), age (18-19, 20-29, 30-39, 40-49, 50-65, 65 and above) and geographical region (village/city). The statistics considered for sampling were based on the national census of 4 December, 2011 [23]. With the guidance of the census, five main regions, namely, Nicosia, Famagusta, Kyrenia, Morfou, and İskele, were examined in terms of the main characteristics of their populations. According to the census data, gender, age and region were divided in to quotas which are arranged according to the general population statistics. The stratification of region, gender, age and quarters/villages/cities were arranged by using the proportionate stratification method as the number of the participants in each region were determined by the census 2011. These five central areas are divided into quarters in the rural area and villages in the urban area. 16 quarters, 17 villages and 5 cities were considered randomly in the study.

### Fieldwork

The fieldwork was conducted from April to June 2016. Starting points were randomly selected in particular streets for cities, and in village centers (coffee houses and village mosques) with directions to the north, south, east and west established for the villages. Interviewers tried to draw squares in their movements, starting with the lowest house numbers. One house in three was added to study with the interviewers taking the first right turning each case in order to complete the square. After one square had been completed, a new start point was defined and the creation of a new square commenced. Gender and age quotas were considered in every house entered. Each pollster considered these quotas in every house. If no one was at house or when participants did not give consent, pollsters continued with the next house. Only one person was added to the study in each house, alternating between men and women. If there was more than one candidate in a home, the one whose birthday was closest was selected. Most recent birthday method allows all household members to have an equal chance of selection under the assumption that births are random [24]. The term “nearest birthday” is referred to the day and month not the year of the birth. As there were age quotas in every region this procedure does not affect dispersion. Households were taken by a random selection method but one adult was taken according to nearest birthday if there is more than one appropriate person according to quotas. Using such a method enables the pollsters to follow up the same procedure. In this study, all participants were not selected on the birthday basis. The nearest birthday was only selected if there were two or more people in the same age group or in the same sex group in a household. It was also aimed to reduce the systematic bias since it is not known how many people will live in each house and how many people will live in the house. 40 interviewers were used, after training about the questionnaire and the interview process. Each interviewer administered 25 questionnaires. In this way, it was hoped to minimize the margin of error that might result from variation in interviewer application. After detailed information was given to the participants, they were asked to sign a consent form signaling their agreement to participate in the study.

### Survey form

A socio-demographic data form consisting of 21 questions was used to collect profile data and the Turkish version of the Beck Depression Inventory (BDI) was used. BDI was first introduced in 1961 by Beck, Ward, Mendelson, Mock and Erbaugh [25], and subsequently underwent revisions in 1978 (BDI-IA) and 1996 (BDI-II) [26].

### Beck depression inventory

The original BDI form consists of 21 questions. Each item is associated with a behavioral characteristic of depression. The 4-degree scale that accompanies the self-evaluation ranges responses from 0 (no symptoms) to 3 (symptoms highly observed) in terms of emotional, cognitive and motivational symptoms in depression [27]. The internal consistency of work in 1978 showed that two BDI forms were equivalently reliable BDI observes emotional, cognitive and motivational symptoms in depression [25]. The Turkish version of BDI also consists of 21 questions. The total score range is between 0 and 63, and the cut-off point is 17, which shows clinical depression A cutoff score of 17 yielded a sensitivity of 50% and specificity of 92%. The Turkish version of the BDI has been validated. In the sample of Turkish validation study of BDI (*n* = 108), the Cronbach’s alpha coefficient was calculated as .91 [26].

### Ethical considerations

The study was approved by the Social and Science Institute Ethical Board at the Near East University of NC and was conducted according to the ethical standards laid down in the 1964 Declaration of Helsinki and its later amendments. Written informed consent from all participants was also obtained.

### Data analysis

In this study, probability weight was used as specifying the sampling design. The probability weight is calculated as N/n, where N = the number of elements in the population and n = the number of elements in the sample. After corrections have been made on the data, all calculations in the study have been made according to sampling weight. In the statistical analyses, Chi-square (×2) analysis was used to compare different sociodemographic characteristics of depressive (BDI ≥ 17) and non-depressive (BDI < 17) participant groups. Statistical significance was defined as *p* < 0.05. Multivariate logistic regression was also used to explore the relation between depression and possible risk factors. SPSS version 23.0 was used for analysis. In the regression model mean scores of demographic variables which have a statistical meaningful difference after the Pearson Chi-square analysis between the depressive and non-depressive participants were included.

## Results

There were 994 participants in the study, but 978 (98%) of the forms were used for statistical analysis as 16 (2%) of them had inconsistent or inconclusive answers. 9 of these forms that are considered as invalid were belonged to female participants and 7 of them were belonged to male participants. 465 (47.4%) of the participants were.

female and 515 (52.6%) male. When the 2011 census was examined, it was found that female (47.4%) and male (52.6%) proportions were similar to the present study. The mean age of the participants with depression was 39.60 ± 16.55, and 39.23 ± 14.81 for the ones without depression (*t* = −0.324, *p* = 0.746). The distribution of the birthplace of the participants was 479 (48.8%) in Cyprus, 449 (45.8%) in Turkey, 13 (1.3%) in the UK and 40 (4.1%) in other countries. With regard to education, 50 (5.1%) were primary school graduates, 146 participants (14.9%) were elementary school graduates, 134 (13.7%) secondary school graduates, 276 (28.1%) high school graduates and 374 (38.2%) university graduates. With regard to marital status, 523 (53.4%) participants were married, 260 (26.5%) single, 64 (6.5%) in relationships, 54 (5.5%) widowed, 46 (4.7%) engaged and 29 (3.0%) divorced. 581 (59.3%) of the participants lived in urban areas, 256 (26.1%) in rural areas and 143 (14.6%) lived in towns.

In terms of the outcomes, 230 (23.5%) of the participants met the criteria for depression, as opposed to 748 (76.5%) did not. Women had a significantly higher rate of depression compared to men. Participants in the 18-29 age groups and in the 50 years and older age group had a significantly higher ratio of depression than the 30-49 age groups. Participants, who were divorced, widowed, engaged or in a relationship had higher depression rates than married and single participants. Participants who were graduates of elementary schools or below had higher rates of depression than participants who were graduates of secondary schools or above. Participants who lived alone had higher rates of depression than those living with a spouse/ partner/ mother/ father/ siblings. Unemployed participants had a higher depression rate than those in employment. It was found that as the monthly income level of the participants decreased, the proportion of depression increased. The highest proportion of depression was found among participants who had no income or were on the minimum wage (1700 Turkish Liras). Participants with physical illnesses had higher depression rates than those without any illness. Participants who used psychoactive drugs also had higher rates of depression, but there was no significant difference for depression rate according to use of alcohol or cigarettes (Table 1).

**Table 1:** Demographics of Depressive Participants (BDI ≥ 17) and Non-Depressive Participants (BDI ˂ 17) participants in North Cyprus

Demographic Variables	Overall sample %	Depressive Participants %	Non-Depressive Participants %	x^2^	p
Gender (*n* = 978)					
Female	47.4	30.1	69.9	20.005	<0.001*
Male	52.6	17.9	82.1		
Age (*n* = 973)					
18-29	30.9	25.9	74.1	3.860	0,145
30-50	43.4	20.4	79.6		
50 and above	25.7	25.7	74.3		
Birth Place (n = 978)					
Cyprus	48.8	21.2	78.8	4.185	0.242
Turkey	45.8	26.7	73.3		
Britain	1.3	23.1	76.9		
Other	4.1	20.0	80.0		
Marital Status (n = 978)					
Married	73.1	19.7	80.3	20.211	<0.001*
Single	15.3	34.5	65.5		
Divorced	4.1	29.6	70.4		
Widow	7.5	40.4	59.6		
Having Children (*n* = 870)					
No Children	40.7	24.9	75.1	0.843	0.359
Have Children	59.3	22.2	77.8		
Living Place (*n* = 977)					
Village	30.6	22.7	77.3	0.032	0.857
City	69.4	23.3	76.7		
Employment Status (n = 977)					
Employed	59.1	17.0	83.0	34.271	<0.001*
Unemployment	40.9	33.2	66.8		
Education Level (n = 978)					
Illiterate	5.1	44.0	56.0	13.780	0.001*
Primary-Secondary School	28.6	25.2	74.8		
High School and above	66.3	21.3	78.7		
Monthly Income (*n* = 975)					
1700 TL and beloved	34.1	32.0	68.0	20.849	<0.001*
1701-10,000 TL	61.7	18.8	81.2		
10,000 TL and more	4.3	26.2	73.8		
Physical Illness (n = 977)					
Have Physical Disease	9.2	36.7	63.3	9.272	0.002*
Doesn’t Have Physical Disease	90.8	22.3	77.7		
Whom Living With (n = 978)					
Alone	13.8	33.3	66.7	19.524	<0.001*
Spouse / Partner / Lover	53.6	19.1	80.9		
Mother / Father / Brother	18.7	22.4	77.6		
Other	13.9	32.8	67.2		
Alcohol Use (*n* = 976)					
Non-user	23.2	24.0	76.0	2.536	0.281
1-40 times	28.3	26.5	73.5		
40 times and above	48.6	21.5	78.5		
Smoking (n = 976)					
Non-user	30.7	21.5	78.5	1.177	0.555
1-40 times	15.6	25.0	75.0		
40 times and above	53.8	24.6	75.4		
Drug Use (n = 978)					
User	30.4	26.0	74.0	6.672	0.010*
Non-user	69.6	18.3	81.7		

*p < 0.05 significant level

The proportions of depression with regard to occupation were as follows: housewives (42.1%), unemployed (33.3%), students (30.8%), freelance (24.3%), civil servants (19.4%), workers (19.5%) and business owners (14.0%) (×2 = 46.679, *p* = 0.000). Participants who had 4 or more children had higher depression proportions (41.1%) than those who had 1 (16.7%) or 2-3 children (19.3%) (× 2 = 22.697, p = 0.000). The number of years the participants had been settled in Cyprus, was found to have no effect on depression rate (×2 = 11.215, *p* = 0.082).

When participants’ expectations of a political solution in Cyprus were evaluated, it was discovered that those who want a bi-zonal bi-communal federal state, and those who want a separate republic as a continuation of NC (18.8%) had lower depression proportions than those desiring a two state confederated state solution (27.1%), a return to the1960 Cyprus Republic (25.8%), or union with Turkey (31.8%). No difference was found between groups according to the ratio of depression in terms of whether their houses are original Greek or original Turkish property (×2 = 4.083, *p* = 0.130), or they own the house or not (×2 = 6.763, *p* = 0.080) (Table 2).

**Table 2:** Ideas about Cyprus Political Solution and Status of Home lived of Depressive Participants (BDI ≥ 17) and Non-Depressive participants (BDI ˂ 17) in North Cyprus

Demographic Variables	Depressive Participants %	Non-Depressive Participants %	x^2^		p
Own Resources (n = 977)					
Owned	21.5	78.5	6.763		0.080
Government Owned	33.9	66.1			
On Rent	26.9	73.1			
Other	20.7	79.3			
Status of Home Lived (*n* = 971)					
Turkish Property	24.6	75.4	4.083		0.130
Greek Property (Allocated)	25.8	74.2			
Greek Property (Equivalent)	17.8	82.2			
Ideas about Cyprus Political Solution (n = 978)					
New Federal state	18.8	81.2	18.865		0.002*
Con-federal states	27.1	72.9			
Continuation of Status	18.0	82.0			
Unite to Turkey	32.0	68.0			
Forming again 1960 Republic of Cyprus	25.8	74.2			

*p < 0.05 significant level

Thus the possible risk factors for depression have been identified as being female, living apart from the family, having an education below high school level, using psychoactive drugs, being unemployed, having an income level less than 3400 Turkish Liras, having a physical disease and wanting a confederated solution in Cyprus (Table 3).

**Table 3:** Odss Ratio and Confidence Intervals of some demographic variables obtaining from Multivariate Logistic regression

Demographic variables	Depressive / Non-Depressive Participants	
	Odss Ratio	95% CI
Gender (Female / Male)	1.422	(1.199 – 1.687)**
Living status (not with family / with family)	1.724	(1.252 – 2.374)**
Education (High school below / above)	1.438	(1.059 – 1.952)*
Drugs (user / not user)	1.382	(1.070 – 1.784)*
Marital Status (single / married)	1.601	(1.190-2.155)*
Employment Status (unemployed / employed)	2.425	(1.795-3.276)**
Monthly Income (3400 TL below / above)	1.602	(1.212-2.118)**
Physical Disease (having/don’t having)	1.686	(1.251-2.271)**
Solution in TRNC (willing con-federal/federal)	1.424	(1.030-1.970) *

*p ≤ 0.05

**p ≤ 0.001 significant level, CI = Confidence Interval

## Discussion

This cross-sectional study found a point prevalence of 23.4% for relatively high BDI scores (≥17) suggesting estimated number of 68.888 people suffering clinical depression among 296.396 people who live in North Cyprus. Being female, a widow, unemployed, having a limited education and low income level, having a physical illness, wanting a confederated solution in Cyprus, living alone, being widowed and using illicit substances were defined as risk factors for depression.

Other international surveys using self-rating scales in community samples have reported prevalence estimates ranging from 11% to 23% [28–32]. The wide range of estimates in the prevalence of depressive symptoms may be the result of the diverse methodological approaches, various diagnostic tools used and socio-cultural characteristics of the different target populations [10]. In epidemiological studies, using different cutoff scores in scales of measuring depression may also result in different prevalence estimates for depression [28]. Nonetheless, extremely high prevalence depression proportions have been found in some specific cultures such as Puerto Rico [33], Native Alaskans and Native Americans [34] and Australian Aboriginal people [35]. These cultures share the common characteristics of a history of colonization and related economic exploitation, low education, self-identity problem, a rise in unemployment, increased prevalence of some chronic diseases and dependence on other communities. Extremely high prevalence depression proportions have been also found in such countries as Afghanistan [36], Honduras and the Palestinian territories [6], India [37], Nepal [38], Brazil [39] and Southwest Ethiopia [40]. War and migration histories, economic hardship, a rise in unemployment and socioeconomic problems are the main reasons that increase depression prevalence. These findings, both in specific cultures and countries, show the common macro or environmental reason that increase depression prevalence. When we consider the world prevalence, it is clear that NC also has one of the higher depression prevalences. The fact that NC is not a recognized country, is dependent economically and politically on Turkey, recent war (in 1974), migration and British colonial history, previous economic crisis, the uncertainty about the Cyprus Problem, high unemployment rates, and corrupt public order may be seen as the reason for the high depression levels. It has been stated in research that war [41], migration [42], economic crisis [43, 44] and unemployment [45, 46] can lead to depression. Previous research conducted in NC also supports these findings. Both North [47] and South Cyprus societies [10] experienced depression as well as PTSD because of war and losing relatives. Ergun et al. (2008) [48] stated that migrants also experienced depression when compared to people who did not migrate as a result of the war. Aktolgalı and Çakıcı (2001) [49] also identified that the economic crisis and bankruptcy of the banks created intense psychological distress and depression, concern about the future and hostility in NC. The deadlock in the Cyprus Problem and future prospects for solution also caused depressive thoughts and emotions [50].

In some studies, racial/ ethnic minorities have been found to suffer from higher prevalence of depression [34, 35]. Other studies however have found similar [40] or even lower prevalence [51] among racial/ethnic minorities. The US Department of Health and Human Services (2001) reported that some minority groups have common possible risk factors for mental illness resulting from the fact that these populations often experience social and economic inequality, exposure to racism and discrimination, increased prevalence of some chronic diseases, and less access to care and treatment for mental and physical health conditions [52]. This study showed that a similar depression prevalence proportion is evident among both native Turkish Cypriots and immigrants from Turkey. Since immigration has mainly taken place post-partition of the island between Greek Cypriots and Turkish Cypriots in 1974, there has been a substantial migration of Turkish people to North Cyprus. As a former British colony, many Turkish Cypriots also immigrated to the UK, as well as to other Common wealth destinations including Australia, South Africa and Canada, as well as The United States. Consequently, the number of immigrants from Turkey has outgrown the number of Turkish Cypriots [53]. After a 40 year period of migration from Turkey, the native Turkish Cypriot population has decreased to about 115,000-85,000 and the Turkish migrant population increased to 130,000-160,000. The social, economic and political relations between native Turkish Cypriots and immigrants from Turkey are strained, with Turkish Cypriots expressing the feeling that they have been colonized and invaded culturally [54]. Experiences of acculturation, changes in sociocultural norms and loss of social or economic status can all be considered reasons for the development of mental health problems [55, 56]. It is also observed however that not every mental health process is influenced by acculturation processes in the same way. While pathological gambling is found to be more common among native Turkish Cypriots than the immigrants from Turkey [57], illicit drug use is more common among immigrants from Turkey [58]. When cultural attitudes of both groups are observed, acculturation strategies of separation chosen by native Turkish Cypriots, and assimilation chosen by immigrants from Turkey were found to be the reasons for high proportions of drug use among immigrants from Turkey [59] and problem and pathologic gambling proportions among native Turkish Cypriots [60]. Hence minority characteristics may impact on certain mental disorder processes, whilst being an immigrant may be the origin of others. Community behavioral reactions that result in different mental disorder prevalences thus depend on each culture’s own unique socio-cultural context and acculturation configurations. In line with this, despite the shared risk factors, there are psychosocial factors that uniquely confer risk to one class of disorders over the other [61]. In other studies of depression also, significant differences have been demonstrated across cultures in the experience, expression and consequences of a range of emotional terms and illnesses [62].

When political views are considered, people who would like to unite with Turkey, support confederation, and want to reform the 1960 Republic of Cyprus again exhibit significantly higher prevalence of depression compared to people who are pleased with the current unrecognized government “Turkish Republic of Northern Cyprus” and people who believe in forming a new government with bi-zonal bi-communal federal states. Political beliefs and housing status are related with the previous war and migration events of the participants living in the NC. The negotiation, which has been ongoing for 40 years between the Turkish Cypriots and the Greek Cypriots in the NC today, causes continuing uncertainty about the future which in turn has an impact on depression. The results of this study show that the Cyprus problem negotiations that have been continuing for some 40 years under the auspices of The United Nations with a view to establishing a united federal government of Cyprus may disappoint people those who believe in a con-federal system, union with Turkey, or a return to the 1960 Republic. Furthermore, the ambiguity of the Cyprus problem, has entrenched the status quo. Studies which focus on the relationship between stress and depression have shown that sociopolitical reasons can be underlying factors in the development of depression [63, 64]. Studies have also shown that depression is also elevated during and after events such as war, prolonged socio-political conflict and political turmoil in low-income countries [65, 66].

According to the research data, the depression prevalence in women is higher than in men. This is consistent with previous research suggesting that depression is more common in women than men [67–72]. However, not all research supports this finding. Studies of people who experienced war in Syria [40] and of a student group who did not graduate in Sri Lanka [73, 74] showed no significant difference between male and female depression prevalence proportions. The higher prevalence of depression levels in women is thus not fully explained. In research carried out so far, it has been found that depression may be caused by women’s hormonal changes. Also, postpartum and premenstrual periods can cause to depression [75].

Even though age groups were not statistically different, the depression prevalence amongst the youth and elderly was higher compared with middle age. Studies suggest that point prevalence in youth and elderly is much higher [10, 76, 77]. The higher prevalence of depression in the elderly can be explained by biological changes, medical problems and older people’s diminished mental activity [78]. On the other hand, hormonal changes [79], low self-esteem [80], and the need to belong [81] may explain the higher prevalence in youth.

When marital status was examined, it was discovered that married people have lower depression prevalence then divorced and widowed. However, Yan’s study in China stated that being married and over 55 of age is a risk factor compared to being divorced or widowed [19]. Being divorced or widowed is a phenomenon influenced by cultural norms [82]. Divorced people usually plan to re-marry, but only some of them do. In NC, divorce is seen as unacceptable by some, especially for women. All these findings show that marital status may have different effects on depression in different cultures. Although studies among Turkish societies in Turkey [83, 84] show a relation between having children and depression level, this was not the case in this present study because of the family structure in NC. Due to the fact that, Turkish Cypriot family structure is an extensive family structure which is crowded and supportive. This kind of a family structure decreases the responsibilities of raising a child as it enables to receive support and share the responsibilities of the children with other family members.

In this study, as with other similar research, individuals living alone [74, 85, 86], unemployed [87, 88], with a low educational level [70, 89], lower incomes [78, 90, 91], or physical illness [70, 90, 92] have higher depression prevalence. Smoking and use of alcohol are not considered as risk factors in the development of depression in NC. Although this finding is also supported by some research [93–95], other studies have found that smoking and alcohol use do indeed have a direct correlation with depression [40, 67–73]. However the depression prevalence in illicit psychoactive substance users compared with non-users is higher. Illicit psychoactive substance use is known to lead to depression [40, 93].

### Limitations

First, the BDI scale, like other self-reporting screening instruments, is not perfect in measuring clinical depression; it is mainly used to assess symptom severity rather than as a diagnostic tool [96]. Second, it is impossible to as certain temporality in cross-sectional studies. For example, having economic problems can lead to depression, but depression can also lead to economic problems. Third, non-response bias may cause underestimation of the levels of depressive symptoms, because people who participate in this kind of health surveys are healthier than those who do not [97]. Fourth, some populations are not included in the samples such as those in prisons, dormitories, hospitals or the army.

## Conclusion

Extremely high prevalence of clinical depression was observed among North Cyprus Population. Being women, living alone, low level of education, being widowed, using drugs are found to be main possible risk factors for depression. In regions like NC, further research with long-term follow-up is needed to increase our understanding of the possible risk factors for extremely high depression prevalence at-risk populations. Each culture or country has unique characteristics specific to itself, besides individual factors socio-cultural features like socioeconomic problems, high unemployment, war, migration and colonization history, prevalence of some chronic diseases and dependence on another country may lead to an increase in the prevalence of depression. North Cyprus Population is faced with many struggles throughout their lives relative to their historical, cultural, and social structural position in Cyprus. Because of these issues heighten NC population’s vulnerability for depressive symptoms, depression must be prior public mental healthcare in NC. It is useful for researchers and policy makers to understand the socio-cultural factors as well as the individual factors behind depression as they seek to evaluate and improve mental health program policy in NC. The prevalence of depressive disorders should be monitored by NC Health Ministry and Public Health Department through constant surveillance, and socio-cultural characteristics also should be considered when planning and implementing interventions. The understanding of the social atmosphere that surrounds the major depressive patients is the focus of consideration of the patients together with socio-cultural factors, not just physical symptoms. Socio-cultural factors are important in the development of major depressive disorder and the evaluation of social factors such as stress, lifestyle, income, unemployment and deprivation will have a positive impact on the recovery process. Understanding of the relationship between socio-cultural factors and major depression, positive changes in social and economic fields as well as individual therapies, and preventive public health policies related to these issues will reduce the prevalence of major depression.

## Additional file

Additional file 1: Weight total - depression-prevalence dataset. (XLSX 258 kb)

## Abbreviations

BDI: Beck Depression Inventory
NC: North Cyprus
SPSS: Statistical Package for the Social Sciences
US: United States
